# COVID-19, Coronavirus, Wuhan Virus, or China Virus? Understanding How to “Do No Harm” When Naming an Infectious Disease

**DOI:** 10.3389/fpsyg.2020.561270

**Published:** 2020-12-09

**Authors:** Theodore C. Masters-Waage, Nilotpal Jha, Jochen Reb

**Affiliations:** Lee Kong Chian School of Business, Singapore Management University, Singapore, Singapore

**Keywords:** psychology of naming, COVID-19, Wuhan Virus, coronavirus, pandemic, public messaging, China Virus, sinophobia

## Abstract

When labeling an infectious disease, officially sanctioned scientific names, e.g., “H1N1 virus,” are recommended over place-specific names, e.g., “Spanish flu.” This is due to concerns from policymakers and the WHO that the latter might lead to unintended stigmatization. However, with little empirical support for such negative consequences, authorities might be focusing on limited resources on an overstated issue. This paper empirically investigates the impact of naming against the current backdrop of the 2019–2020 pandemic. The first hypothesis posited that using place-specific names associated with China (e.g., Wuhan Virus or China Virus) leads to greater levels of sinophobia, the negative stigmatization of Chinese individuals. The second hypothesis posited that using a scientific name (e.g., Coronavirus or COVID-19) leads to increased anxiety, risk aversion, beliefs about contagiousness of the virus, and beliefs about mortality rate. Results from two preregistered studies [*N_(Study 1)_* = 504; *N_(Study 2)_* = 412], conducted across three countries with the first study during the early outbreak (April 2020) and the second study at a later stage of the pandemic (August 2020), found no evidence of any adverse effects of naming on sinophobia and strong support for the null hypothesis using Bayesian analyses. Moreover, analyses found no impact of naming on anxiety, risk aversion, beliefs about contagiousness of the virus, or beliefs about mortality rate, with mild to strong support for the null hypothesis across outcomes. Exploratory analyses also found no evidence for the effect of naming being moderated by political affiliation. In conclusion, results provide no evidence that virus naming impacted individual’s attitudes toward Chinese individuals or perceptions of the virus, with the majority of analyses finding strong support for the null hypothesis. Therefore, based on the current evidence, it appears that the importance given to naming infectious diseases might be inflated.

“Having a name matters to prevent the use of other names that can be inaccurate or stigmatizing.”

—Tedros Ghebreyesus, Director-General, World Health Organization (WHO, 2020a).

“We’ve seen certain disease names provoke a backlash against members of particular religious or ethnic communities, create unjustified barriers to travel, commerce and trade, and trigger needless slaughtering of food animals. This can have serious consequences for people’s lives and livelihoods.”

—Keiji Fukuda, Assistant Director-General, World Health Organization (WHO, 2020c).

## Introduction

In the face of a pandemic, one of the key decisions that scientists and policymakers face is how to name the infectious disease. While this decision might seem mundane relative to other urgent matters, international bodies, such as the World Health Organization (WHO) have expressed concern about potential unintended negative consequences of disease names (WHO, 2020a,b). The primary concern is that place-specific names, such as “Spanish Influenza” or “Middle East Respiratory Syndrome” will lead to the stigmatization of individuals associated with this region (WHO, 2020a). Thus, in 2015, the WHO released a report listing what they see as best practices for naming new human infectious diseases to “minimize the unnecessary negative impact of disease names” (Fukuda et al., 2015; WHO, 2020b). However, it generally takes significant deliberation for the WHO to officially sanction a name, by which point alternative names often have arisen in the public lexicon.

The 2020 pandemic is a perfect example of this name multiplicity, with several different monikers emerging. The first name that unofficially started floating around in the media since December 2019 is *Wuhan Virus*. This is a place-specific name derived from the likely emergence of the virus in Wuhan, China. As mentioned above, WHO guidelines warn against such place-specific names (Fukuda et al., 2015; WHO, 2020b), and this name has received negative media attention for its possible impacts on stigmatization and xenophobia (Board, 2020; Gabbatt, 2020). The second name to emerge was *Coronavirus*, a scientific but technically “inaccurate” name referring to the family of viruses. Nevertheless, this continues to be the most popular name in Google search trends (Google Trends, 2020). A third name, “COVID-19,” was released by WHO on February 11, 2020, in line with its guidelines (WHO, 2020b)^1^. Since then, the WHO, many governments, and media outlets have actively sought to instill this name in the public discourse. A fourth name considered is “China Virus” (WHO, 2020a). Similar to Wuhan Virus, this name has been criticized in the media for its potential to promote xenophobia and official briefings using this name have later been retracted (Trump and Donald, 2020). However, despite the rich media discussion, there is little empirical evidence on the psychological impacts of virus naming. To help address this question, we investigate the effects of names on people’s perceptions including sinophobia, anxiety, risk aversion, and mortality and contagiousness beliefs.

In the case of the current pandemic, the primary contrast is between the scientific names (COVID-19 or Coronavirus) and the place-specific names (Wuhan Virus or China Virus). Empirical research suggests that names play an important role in how we perceive phenomena (Wood, 1991; Waytz et al., 2014), although findings have been somewhat mixed. For example, in the health domain, studies have found that both drug (e.g., “opioid” vs. “narcotic”) and illness names (e.g., “gout” vs. “urate crystal arthritis”) significantly impact patient and public reactions (Mangione and Crowley-Matoka, 2008; Petrie et al., 2018). However, in the domain of naming natural disasters, evidence has been inconclusive, with initial findings suggesting that female-named hurricanes led to significantly more deaths because they were erroneously perceived as less dangerous (Jung et al., 2014), but a reanalysis of the data found no support for this naming effect (Malter, 2014). Thus, the psychological effect of naming is very much an open topic for research.

The first research question this paper investigates is whether using a place-specific name leads to increased xenophobia toward individuals from that country. As discussed, the names Wuhan Virus and China Virus are generally shunned in media circles, and their use has been anecdotally linked to acts of violence against ethnically Chinese individuals living abroad (Al Jazeera, 2020; Board, 2020; Gabbatt, 2020). Psychologically, this is attributed to a process by which individuals associate their negative views toward the pandemic with a specific population (i.e., Chinese) and subsequently develop negative views about that population (Fukuda et al., 2015). We empirically test this possibility, exploring the effects of naming on *sinophobia*, the negative stigmatization of Chinese individuals. More specifically, if the above reasoning is correct, we would expect to find more negative views of Chinese people (i.e., sinophobia) when the pandemic is referred to by a place-specific name, i.e., Wuhan Virus or China Virus.

Moreover, we examine whether political affiliation moderates this naming effect. The theoretical rationale for such a moderation lies in political affiliation being related to ingroup favoritism, with conservatives showing stronger ingroup bias than liberals during times of threat (Perry et al., 2018). This ingroup favoritism could lead to increased sinophobia, specifically when the pandemic is referred to by place-specific names.

The second research question this paper investigates is the potential negative effect of using the official scientific name on attitudes toward the pandemic. Research has found that scientific concepts can lead to greater feelings of stress and increased aversion (Mallow, 1994). Further, scientific names are also generally not in the common lexicon and devoid of any human association, which could result in individuals feeling greater distrust of the phenomenon (Waytz et al., 2014). If this reasoning is correct, we would expect to find more negative perceptions of the pandemic when it is referred to with its scientific name, COVID-19 or Coronavirus, as compared to the place-specific names.

In sum, this paper tests two hypotheses with respect to naming: (a) that the place-specific names (Wuhan Virus/China Virus) lead to increased sinophobia relative to other names (Hypothesis 1) and (b) that the scientific names (COVID-19/Coronavirus) lead to more negative attitudes—in the form of increased levels of anxiety, risk aversion, and beliefs about contagiousness and mortality—relative to other names (Hypothesis 2). Also, we explore political affiliation as a potential moderator of this effect. Two separate studies were conducted during the early outbreak of the pandemic (April 2020) and at its later stages (August 2020). The entire set of study materials and analysis plans for both studies were pre-registered before data collection (see osf.io/9s4jk). Given the global nature of the pandemic, we collected data from three countries—US, Canada, and India—in Study 1 (*N* = 504) investigating the names Wuhan Virus, COVID-19, and Coronavirus and two countries—US and India—in Study 2 (*N* = 412) adding the name China Virus.

## Study 1

### Materials and Methods

In the first study, we obtained three samples from the United States of America (US), Canada, and India. All participants were recruited through the online surveying platform Amazon Mechanical Turk. Following the best practices in ensuring participant quality (Keith et al., 2017), we screened for participants who (a) had completed at least 50 previous surveys and (b) had a past participant approval rating of 95% and above.

A demographic breakdown across the total sample (*N* = 504) shows a mean age of 36.09 (*SD* = 10.71), 29.96% female, 43.06% Caucasian (38.69% Indian, 7.34% Black, 1.98% Chinese, 8.93% Other), and 52.18% having an undergraduate degree (14.09% lower qualifications and 33.73% higher qualifications).

The study procedure was identical across all three samples. Participants first read an article describing the spread of the pandemic and then answered questions relating to (a) state anxiety, (b) domain-specific risk aversion, (c) beliefs about contagiousness and mortality of the virus, and (d) attitudes toward Chinese individuals.

**FIGURE 1:**
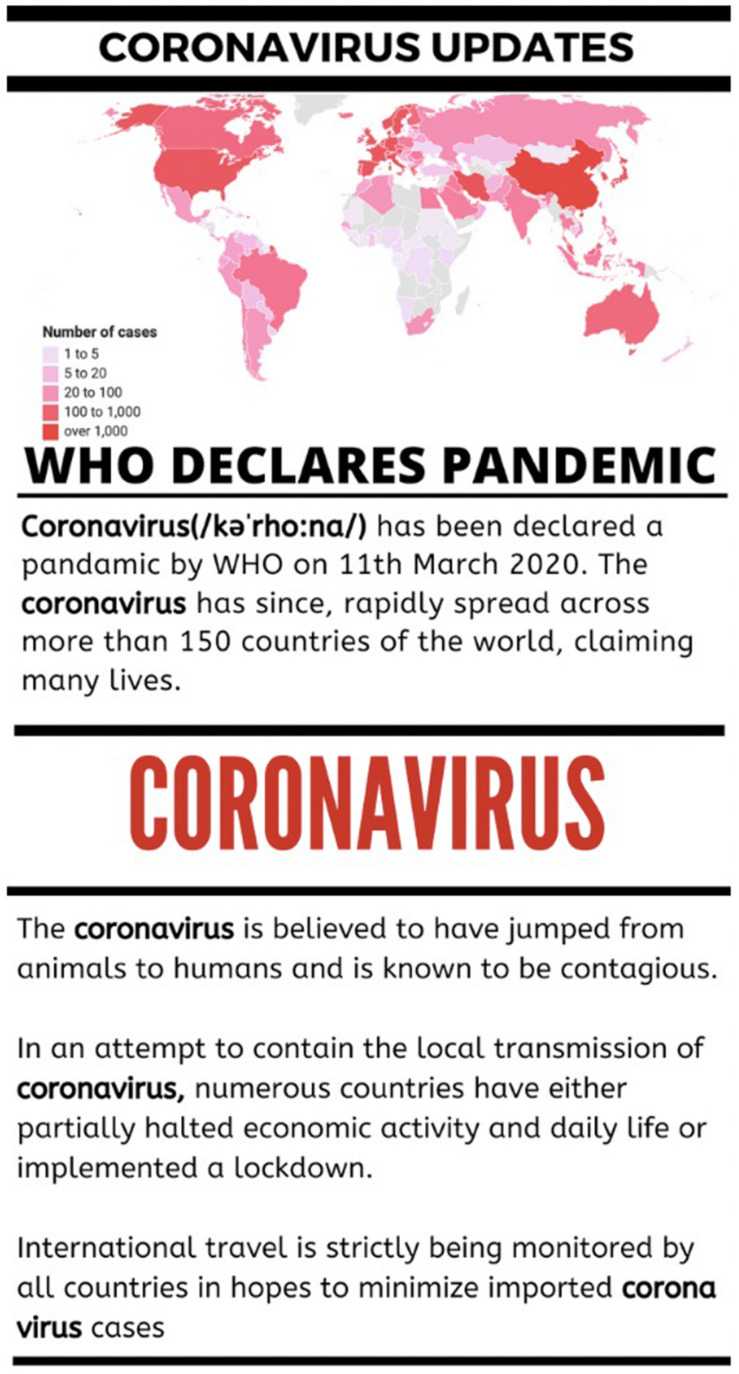
Presented below is an example of the article used to manipulate the name of the virus (in this case “coronavirus”).

We manipulated one factor, virus name, across three levels: COVID-19, Coronavirus, and Wuhan Virus. We did so by using the respective name in the article (an example is shown in Figure 1) and in the following questions mentioning the virus (e.g., “Out of 100 people who are infected with the (COVID-19, Coronavirus, Wuhan Virus) how many do you think will die as a result of catching the virus?”). More details on the methods, manipulations, measures, pre-registered exclusions, and analysis plan are available on OSF (see osf.io/9s4jk).

### Measures

#### Anxiety

We measured state anxiety after reading the article using the PANAS-X fear subscale (Watson and Clark, 1999). Participants rated how well five different emotion words (nervous, scared, frightened, jittery, and shaky) characterized their current emotional state on a scale from 1 (“strongly disagree”) to 7 (“strongly agree”) [Cronbach’s alpha (α) = 0.90].

#### Domain-Specific Risk

We used an adaptation of the DOSPERT scale (Blais and Weber, 2006) with scenarios that relate specifically to the current pandemic to measure risk aversion. This scale (see Appendix) attempted to capture perceived risk related to different activities in the time of the pandemic. The scale demonstrated reasonable internal consistency (α = 0.85).

#### Beliefs About Contagiousness and Mortality

We used two one-item measures developed by Fetzer et al. (2020) to measure beliefs about (a) how contagious the virus was and (b) how many out of 100 people infected would die from the virus. Answers ranged from 0 to 100 on both scales. Akin to the original paper, these responses were heavily skewed and thus all responses were logged (Fetzer et al., 2020).

#### Sinophobia

To measure prejudice toward Chinese individuals, we adapted an explicit measure developed by Payne et al. (2010) to measure prejudice against black individuals. This included a measure of perceived warmth along with feelings of admiration and sympathy. Items were combined to form a single measure of sinophobia (α = 0.68). We opted for an explicit measure, instead of an implicit measure, based on findings that explicit measures provide adequate assessments of prejudice (Axt, 2018). This measure was standardized with positive scores indicating sinophobia.

#### Political Affiliation

Political affiliation was measured across all samples using a one-item five-point self-reported measure developed by McAdams et al. (2008), which asked participants: “How would you define yourself on the following scale in terms of your political orientation?” (1 = very liberal, 2 = liberal, 3 = middle of the road, 4 = conservative, and 5 = very conservative). This measure was then simplified into a categorical variable to create a clearer contrast between liberal (1) vs. middle of the road (2) vs. conservative (3).

#### Education Level

Level of education was measured based on the highest level of qualification received by the participant: high school diploma, bachelor’s degree, and postgraduate degree (master’s/doctoral degree). This was coded as a categorical variable (1–3).

#### Age and Gender

Demographic variables were measured using single items for age (18–100 +), gender (female = 0; male = 1), and education level (high school diploma = 1; bachelor’s degree = 2; postgraduate degree = 3).

### Exclusions

Exclusions were applied in line with the OSF pre-registration. First of all, given the relatively subtle nature of the intervention, we excluded participants who failed an instructional manipulation check (Oppenheimer et al., 2009). Second, given the potential impacts on the outcome variables of interest, we excluded individuals who (a) had the virus, (b) were in physical contact with someone who had the virus, or (c) had close family and friends who had the virus. These exclusions did significantly cut the sample size (a total of 245 participants were excluded).

### Analysis Plan

We followed the analysis plan in line with the OSF pre-registration. We compared means across conditions (COVID-19 vs. Coronavirus vs. Wuhan Virus) using analysis of variance (ANOVA). We followed up with Bayesian analyses to evaluate the null hypothesis of no naming effect. Please note that all the Bayesian factors reported in this paper compare the likelihood of the data occurring under the alternative hypothesis vs. the null hypothesis (BF_10_). For example, a Bayes Factor of 10.00 indicates that the data are 10 times more likely to occur under the alternative hypothesis compared to the null hypothesis; alternatively, a Bayes Factor of 0.1 indicates that the data are 10 times more likely to occur under the null hypothesis compared to the alternate hypothesis (Jarosz and Wiley, 2014). These analyses were conducted using JASP with a standard unbiased Cauchy prior using the JASP default width of 0.5 (JASP Team, 2020). See Etz and Vandekerckhove (2018) and Wagenmakers et al. (2018) for more information on interpretation for Bayes analyses. Finally, exploratory analyses, i.e., not formally pre-registered, explored the moderating effects of political affiliation.

### Results

Descriptive statistics and correlations are provided in Table 1.

**TABLE 1:** Summary statistics and correlations for Study 1. Presented below are the means, standard deviations, and correlations for all variables across the entire sample.

	M	S.D.	Min	Max	1	2	3	4	5
Anxiety	2.99	1.11	1	5	[0.90]				
Risk aversion (DOSPERT)	5.50	0.92	1	7	0.43***	[0.85]			
Contagious beliefs _(logged)_	2.47	1.13	0	4.61	0.25***	0.27***			
Mortality beliefs_(logged)_	2.02	1.03	0	4.61	0.35***	0.23***	0.34***		
Sinophobia	0.17	0.97	−2.00	2.33	−0.04	0.12**	0.19***	−0.04	[0.68]
Political affiliation	1.97	0.88	1	3	0.22***	−0.01	−0.04	0.12**	–0.07

Alpha coefficients for composite measures are provided in brackets. ***p < 0.001, **p < 0.01.

#### Sinophobia

We compared means across conditions for sinophobia using the three measures outlined by Payne and colleagues (Payne et al., 2010): warmth, admiration, and sympathy. We found no differences across conditions on sinophobia, with strong support for the null hypothesis [*F*(2, 501) = 0.78, *p* = 0.46, η = 0.00; *Bayes Factor_(BF10)_* = 0.047]. In sum, in evaluating Hypothesis 1, there was strong evidence for the null hypothesis, i.e., place-specific naming did not increase sinophobia.

Next, we conducted exploratory analyses to see if any differences emerged depending on political affiliation (liberal vs. conservative; *M* = 1.96, *SD* = 0.89). Analyses found no evidence of a significant interaction with condition with very strong support for the null hypothesis, *F*(4, 489) = 0.71, *p* = 0.59, η = 0.01; *BF*_10_ = 0.000842. This suggests that political affiliation did not moderate sinophobic responses to different virus names.

#### Anxiety, Risk Aversion, and Beliefs About the Virus

First, we compared means across the three naming conditions for anxiety, risk aversion and beliefs about contagiousness/mortality separately. Below, we report results pooled across the three samples (US, Canada, and India); note that similar patterns were seen within country samples (see Table 2). We found no main effect of naming on anxiety, with Bayesian analyses showing very strong support for the null hypothesis, *F*(2, 501) = 0.05, *p* = 0.95, η = 0.00; *BF*_10_ = 0.023. Similarly, no significant differences emerged for the measure of domain-specific risk aversion with mild support for the null, *F*(2, 501) = 1.77, *p* = 0.17, η = 0.01; *BF*_10_ = 0.119. Next, we detected a marginal effect on beliefs about contagiousness although Bayesian analyses still found weak support for the null, *F*(2, 501) = 2.66, *p* = 0.07, η = 0.01; *BF*_10_ = 0.271. Finally, there was no support for an effect on mortality beliefs, with very strong support for the null, *F*(2, 501) = 0.03, *p* = 0.97, η = 0.00; *BF*_10_ = 0.023.

**TABLE 2:** Effect of naming on individual perceptions and beliefs for Study 1. Presented below are the results of one-way ANOVAs run on each of the study variables across the three samples and the entire sample.

Sample	Statistics	Anxiety	Risk aversion (DOSPERT)	Contagiousness beliefs_(logged)_	Mortality beliefs_(logged)_	Sinophobia	No. of observations (N)
US	df	2	2	2	2	2	212
	*F*	0.31	2.22	1.89	0.16	1.83	(77, 72, 63)
	Prob > *F*	0.73	0.11	0.15	0.85	0.16	
	η	0.00	0.02	0.02	0.00	0.02	
Canada	df	2	2	2	2	2	98
	*F*	0.19	0.35	0.08	0.28	0.49	(33, 34, 31)
	Prob > *F*	0.83	0.70	0.92	0.77	0.62	
	H	0.00	0.01	0.00	0.00	0.01	
India	df	2	2	2	2	2	194
	*F*	0.38	1.45	1.52	0.32	0.45	(67, 66, 61)
	Prob > *F*	0.68	0.24	0.22	0.72	0.64	
	η	0.00	0.01	0.01	0.00	0.00	
Overall	df	2	2	2	2	2	504
	*F*	0.05	1.77	2.66	0.03	0.78	(177, 172, 155)
	Prob > *F*	0.95	0.17	0.07	0.97	0.46	
	η	0.00	0.01	0.01	0.00	0.00	

*Brackets under N show no. of observations per manipulation condition (COVID, Coronavirus, Wuhan Virus).*

## Study 2

The result of the first study provided consistent support for a null effect of virus naming on sinophobia and attitudes toward the virus. To further corroborate these results, which ran counter to our pre-registered hypotheses, a follow-up study was conducted. The aims of this study were twofold. First, the study sought to address whether the impacts of naming perhaps only emerge after increased exposure to all of the names by examining the same hypotheses at a second time point much later after the initial outbreak (August 2020). Additionally, given the null effects of the place-specific name “Wuhan Virus” on impacting sinophobia, we sought to investigate whether using a name more explicitly linking China with the pandemic, i.e., “China Virus,” might impact sinophobia. As in the previous study, all materials and analysis plans were pre-registered at osf.io/9s4jk.

### Materials and Methods

In the second study, we obtained samples from the US and India. All participants were again recruited through the online surveying platform Amazon Mechanical Turk with the same pre-qualifications as in Study 1 for ensuring participant quality. A demographic breakdown across the total sample (*N* = 412) shows a mean age of 37.63 (*SD* = 12.48), 37.62% female, 48.06% Caucasian (39.32% Indian, 7.04% Black, 1.21% Chinese, 4.37% Other), and 58.01% having an undergraduate degree (14.08% lower qualification and 27.91% higher qualification).

The study procedure was identical to Study 1 with the addition of another moniker (China Virus) as the fourth experimental condition and a different measure of coronavirus-specific risk perceptions. At the time of conducting this second study, a scale for coronavirus specific risk perceptions had been validated by Dryhurst et al. (2020). Therefore, we decided to opt for the validated measure to provide consistent evidence across two scales and use a psychometrically valid scale. A forced response type manipulation check at the end of the survey asked participants to report the name of the virus as seen in the manipulation. A chi-square test indicated a significant relationship between the manipulation check and the manipulated names [χ^2^(12, *N* = 412) = 1059.12, *p* < 0.001], indicating that most people gave the correct response and that the manipulation was effective. After the study, participants were debriefed and thanked for their participation.

### Measures

All measures from Study 1 were included in this study and multi-item scales showed similar internal consistencies (anxiety, α = 0.94; sinophobia, α = 0.68).

#### Coronavirus-Related Risk Perceptions

A modified version of a coronavirus related risk perceptions scale (Dryhurst et al., 2020) was included as an additional measure of risk perceptions. The scale had six items related to worries relating to the virus [e.g., “How likely is it that you will be directly and personally affected by (virus name) in the next 6 months?”] on a scale from 1 (“strongly disagree”) to 7 (“strongly agree”) (α = 0.65).

### Exclusions

Similar to the first study, we excluded 103 participants who failed an instructional manipulation check. However, in contrast to the first study, we did not exclude 149 individuals who (a) had the virus, (b) were in physical contact with someone who had the virus, or (c) had close family and friends who had the virus^2^. This was to capture the reality of how widespread the virus had become by the time this second study was conducted (August 2020).

### Analysis Plan

The analysis plan remained unchanged from the first study and we compared means across conditions (COVID-19 vs. Coronavirus vs. Wuhan Virus vs. China Virus) using analysis of variance (ANOVA). Similar to Study 1, exploratory analyses explored the moderating effects of political affiliation.

### Results

Descriptive statistics and correlations are provided in Table 3. A comparison of means across each country sample is shown in Table 4. A summary of means across the two studies and four conditions is shown in Table 5.

**TABLE 3:** Summary statistics and correlations for Study 2. Presented below are the means, standard deviations, and correlations for all variables across the entire sample.

	M	S.D.	Min	Max	1	2	3	4	5
Anxiety	2.72	1.22	1	5	[0.94]				
Risk aversion	2.18	0.74	1	5	0.42***	[0.65]			
Contagious beliefs_(logged)_	2.16	0.98	0	4.62	0.20***	0.11*	–		
Mortality beliefs_(logged)_	1.84	0.98	0	4.60	0.33***	0.18***	0.38***		
Sinophobia	0	0.78	−1.68	1.78	−0.16***	−0.20***	0.03	−0.12*	[0.68]
Political affiliation	2.06	0.89	1	3	0.21***	0.12*	0.05	0.15**	0.03

Alpha coefficients for composite measures are provided in brackets. ***p < 0.001, **p < 0.01, *p < 0.05.

**TABLE 4:** Effect of naming on individual perceptions and beliefs for Study 2. Presented below are the results of one-way ANOVAs run on each of the study variables across the two samples and the entire sample.

Sample	Statistics	Anxiety	Risk aversion	Contagiousness beliefs_(logged)_	Mortality beliefs_(logged)_	Sinophobia	No. of observations (N)
US	df	3	3	3	3	3	240
	*F*	0.51	0.52	0.14	0.54	0.52	(58, 60, 60, 62)
	Prob > *F*	0.68	0.67	0.93	0.66	0.67	
	η	0.01	0.01	0.00	0.01	0.01	
India	df	3	3	3	3	3	172
	*F*	1.11	0.23	1.48	5.52	0.76	(39, 42, 44, 47)
	Prob > *F*	0.34	0.88	0.22	>0.01	0.52	
	η	0.02	0.00	0.02	0.09	0.01	
Entire Sample	df	3	3	3	3	3	412
	*F*	0.77	0.59	0.58	2.11	1.22	(97, 102, 104, 109)
	Prob > *F*	0.51	0.62	0.63	0.10	0.30	
	η	0.00	0.00	0.00	0.01	0.01	

*Brackets under N show no. of observations per manipulation condition (COVID, Coronavirus, Wuhan Virus, China Virus).*

**TABLE 5:** Summary of outcome variables in the four conditions.

				Means (*SD*)			
Outcomes	Study	Min	Max	COVID-19	Coronavirus	Wuhan Virus	China Virus
Anxiety	Study 1	1.00	5.00	3.00 (1.09)	2.97 (1.12)	2.99 (1.12)	
	Study 2	1.00	5.00	2.85 (1.28)	2.67 (1.14)	2.61 (1.16)	2.77 (1.27)
Risk aversion	Study 1^1^	1.50	7.00	5.60 (0.83)	5.47 (0.92)	5.42 (1.01)	
	Study 2	1.00	5.00	3.42 (0.76)	3.51 (0.75)	3.38 (0.64)	3.44 (0.68)
Sinophobia	Study 1	-2.00	2.33	0.24 (0.94)	0.16 (0.96)	0.11 (1.01)	
	Study 2	-1.68	1.78	-0.05 (0.80)	-0.07 (0.80)	0.00 (0.75)	0.11 (0.76)
Contagiousness_(logged)_	Study 1	0.00	4.61	2.61 (1.14)	2.33 (1.06)	2.44 (1.16)	
	Study 2	0.00	4.61	2.08 (0.92)	2.20 (0.95)	2.11 (0.97)	2.23 (1.07)
Mortality_(logged)_	Study 1	0.00	4.61	2.04 (1.03)	2.01 (1.01)	2.02 (1.06)	
	Study 2	0.00	4.59	1.78 (0.83)	1.81 (0.92)	1.71 (0.93)	2.03 (1.18)

*^1^(DOSPERT).*

#### Sinophobia

Replicating the results of Study 1, but extended to the new China Virus condition, we found no differences across conditions on sinophobia, with strong support for the null hypothesis [*F*(3, 408) = 1.21, *p* = 0.30, η = 0.01, *BF*_10_ = 0.051] (see also Table 4). Additionally, replicating the results of the first study, the interaction of political affiliation with condition was not significant, with very strong support for the null hypothesis, *F*(6, 399) = 0.56, *p* = 0.76, η = 0.01; *BF*_10_ = 0.002.

#### Anxiety, Risk Aversion, and Beliefs About the Virus

Similar to Study 1, we first compared means across the four naming conditions for anxiety, risk aversion, and beliefs about contagiousness/mortality separately. Below, we report results pooled across the two samples (US and India). Note that similar patterns were seen within place-specific samples (see Table 4). We found no main effect of naming on anxiety, with Bayesian analyses showing “very strong” evidence for the null hypothesis, *F*(3, 408) = 0.77, *p* = 0.51, η = 0.01; *BF*_10_ = 0.028. Similarly, no significant differences emerged for the measure of the risk perceptions with strong support for the null, *F*(3, 408) = 0.59, *p* = 0.62, η = 0.00; *BF*_10_ = 0.022. Similarly, no effects were found on beliefs about contagiousness or mortality with Bayesian analyses, suggesting strong and moderate support for the respective nulls [Contagiousness: *F*(3, 408) = 0.58, *p* = 0.63, η = 0.00; *BF*_10_ = 0.022; Mortality: *F*(3, 408) = 2.11, p = 0.10, η = 0.01; *BF*_10_ = 0.168].

## Discussion

Governments, policymakers, and international bodies must decide how to refer to an infectious disease. As such, significant amounts of effort and consideration go into the process of naming an infectious disease including guidelines being made and international conferences held (WHO, 2020b). Further, academic articles are written about best practices to “do no harm” (Fukuda et al., 2015; WHO, 2020a) and debates are sparked from global media to dinner tables as individuals condemn others for using “incorrect” and “inappropriate” names (Board, 2020; Gabbatt, 2020). However, how necessary are such debates? The present study found no evidence that the use of place-specific names leads to negative attitudes toward individuals from this location (i.e., sinophobia) and, further, Bayesian analyses found strong support for the null hypothesis. This is notable given that potential to cause xenophobia is one of the primary reasons given for not using place-specific names for infectious diseases (Fukuda et al., 2015; WHO, 2020a,b). Additionally, we found no evidence that naming alters anxiety, risk perceptions, or beliefs about the virus. These two empirical results, replicated across two studies at different time points, shed light on the limited impact of infectious disease naming in times of a pandemic and are further discussed below.

The null effect of using a place-specific name (“Wuhan Virus” or “China Virus”) on xenophobia is striking and contrary to the prevalent assumption in public policy discourse and the media (Board, 2020; Gabbatt, 2020; WHO, 2020a,b). In addition, the replication of this effect across two time points and different political affiliations lends robustness to the findings. This result does not imply that a negative association of China with the pandemic does not lead to sinophobia. Instead, it is evidence that the use of place-specific names is not sufficient to generate this negative association. To understand this better, we consider more closely how infectious disease names arise and situate the finding within the literature on racist language use.

The initial name for an infectious disease is typically one that is associated with its location of origin. A similar trend is seen across several infectious diseases, e.g., Spanish Flu, Middle-East Respiratory Syndrome, Zika, or Ebola. This is likely due to the origin being salient in the early outbreak, making it an easy name to generate for the public discourse (WHO, 2020a). There is also a broader tradition of naming phenomena by the place or person of origin that pervades much of our language, for example, names (e.g., O’Reilly, Tang, and Romanov), food (e.g., Kobe beef and English mustard), and species (e.g., Florida panther). For this article, the pertinent question is whether the act of naming an infectious disease by its location (e.g., Wuhan) is enough to create a negative association with people from that location (e.g., Chinese individuals). The results of this paper provide empirical evidence that this may not be the case. Specifically, Bayesian analyses lend strong support to the null hypothesis that using a place-specific name (e.g., Wuhan Virus or China Virus) does *not* lead to increased sinophobia.

These findings speak to a broader literature on the use of racist language in public discourse. Increasing scholarly attention has been given to the impact of racist language since the rise of social media (e.g., Twitter), which gives racist individuals a platform to share and spread their views online (Chaudhry, 2015; Matamoros-Fernández, 2017). The defining element of racism is the act of discrimination against certain individuals or groups (Dovidio, 1986). Past research has documented the negative effect of discriminatory language both on the individuals being discriminated against and the broader society exposed to these terms (Gerstenfeld et al., 2003; Faulkner and Bliuc, 2016). Based on this, the current article suggests that with respect to the naming of infectious diseases, the sole use of place-specific names is not sufficient to incite racist attitudes among the public. Nevertheless, these findings do not speak to a situation in which these terms are used to intentionally associate blame or discriminate against individuals from these locations. Moreover, this research does not consider the important element of how individuals from these locations (e.g., China) feel about the use of the terms and the potential negative psychological impact it might have on these individuals (Mays et al., 2007). Such research would be particularly important so that empirical research can inform social media sites on whether place-specific names should be considered harmful language and thus appropriately moderated (Chaudhry, 2015). In sum, while this research provides the first empirical evidence that infectious disease naming does not impact xenophobia, there are many important avenues for future research to explore.

A second empirical finding from this research is the lack of evidence for an effect of naming on anxiety, risk perceptions, or beliefs about the virus. Further, Bayesian analysis showed mild to strong evidence for the null hypothesis across all outcomes. This finding is one of the few cases showing a null effect of naming on psychological outcomes (cf. Malter, 2014). Instead, reviewing the psychology of naming literature, one would generally find evidence supporting an effect of naming (e.g., Wood, 1991; Jung et al., 2014; Waytz et al., 2014). This is potentially due to a publication bias in psychology favoring significant results over null results (Ferguson and Brannick, 2012; Laws, 2013). A negative consequence of this bias is that it may lead to the false impression that the impact of naming is “always” significant and thus likely pervasive across many different domains. This in turn might have contributed to the strong media and policy discourse around the naming of infectious diseases (Board, 2020; Gabbatt, 2020; WHO, 2020a). Therefore, given the lay hypothesis that naming significantly impacts individual’s perceptions and responses to an infectious disease, the significant evidence in favor of the null hypothesis provided by this paper can be seen as an important contribution to research on the psychology of naming.

This research should be viewed in light of its strengths and limitations, which also point to future research directions. As a strength, the pre-registration of the study materials and analysis plan reduced researcher degrees of freedom, strengthening the paper’s conclusions relating to the main effects of naming (Nosek and Lakens, 2014). Also, the use of Bayesian testing of the null hypothesis helps in providing novel insights into what policymakers and researchers can *decrease* their focus on, as opposed to the general recommendations of what they should *increase* their focus on (Etz and Vandekerckhove, 2018).

Moreover, one important issue in research on the psychology of naming is tracking the impact of long-term exposure to the different names of an infectious disease. It is plausible that repeated exposure to place-specific names, such as “Wuhan Virus” might increase the chance that the negative associations with the pandemic are translated into negative attitudes toward individuals from Wuhan or China more broadly, especially in the light of significant economic impacts of the pandemic on individuals. This paper sought to partially address this issue by replicating the results in a second study conducted nearly 5 months after the initial study. This replication at least demonstrates that the effects are robust to increased exposure to all the names. Nevertheless, future research can explore more specifically the effects of increased exposure to a specific name.

A limitation of this research is its narrowed focus. Given that, to our knowledge, this is the first empirical study investigating the effects of naming during a pandemic, many different topics could have been chosen. We chose to focus on one topic that has gained a lot of media and policy attention, i.e., the potential for harm when naming an infectious disease (Fukuda et al., 2015; Board, 2020; Gabbatt, 2020; WHO, 2020a). However, there are still numerous topics to cover within this domain. Particular areas of interest based on this paper’s findings would be investigating if place-specific names have a negative psychological impact on individuals from those regions (e.g., China). It is plausible that the use of the name Wuhan or China Virus makes Chinese individuals feel villainized or impacts *their* beliefs about the pandemic. Additionally, future research could investigate whether the tone/intention with which the name is used has an impact on the “harm” it causes. In this paper, we focused on a more prosaic use of the names, but it is possible that the name “China Virus” takes on another meaning when it is used by an individual seeking to incite sinophobia.

To conclude, this paper provided the first empirical test of the psychological effects of infectious disease naming. The key takeaway is that naming did not impact levels of sinophobia or anxiety, risk perceptions, and beliefs about the pandemic. Therefore, returning to the goal of “First Doing No Harm” (Fukuda et al., 2015), governments, media outlets, and international bodies can be more assured that their choice of name for an infectious disease is unlikely to lead to harmful xenophobia or negative psychological impacts, and thus they might be best served to focus their limited resources elsewhere.

## Data Availability Statement

The datasets generated for this study can be found in the online repositories. The names of the repository/repositories and accession number(s) can be found below: osf.io/9s4jk.

## Ethics Statement

The studies involving human participants were reviewed and approved by the Singapore Management University Ethics Committee. The patients/participants provided their written informed consent to participate in this study.

## Author Contributions

All authors contributed to the methodological design, data analysis, and writing of the manuscript.

## Conflict of Interest

The authors declare that the research was conducted in the absence of any commercial or financial relationships that could be construed as a potential conflict of interest.

## Footnotes

1 While COVID-19 is technically the name for the disease caused by the virus called SARS-CoV-2, WHO prefers to refer to the virus as “the virus responsible for COVID-19” or “the COVID-19 virus” to avoid the unintended hostility toward certain populations in Asia (Hong Kong is referred to “HKSAR” or Hong Kong Special Administrative Region).

2 We declared in our pre-registration that we would exclude infected individuals and therefore we ran the same set of analyses on the reduced sample as well. The results remained unchanged.

## Appendix

Below is the *ad hoc* measure developed to assess domain specific risk perceptions (Blais and Weber, 2006). For each of the following statements, please indicate the risk you perceive the described activity or behavior to be given the current outbreak of (COVID-19/Coronavirus/Wuhan Virus).

The scale ranged from 1 “Not at all risky” to 7 “Extremely risky.”

Items:

1. Going to a supermarket to buy food
2. Commuting to work on a busy train
3. Traveling on a commercial airplane
4. Going to a bar where there have been no recorded cases of (COVID-19/Coronavirus/Wuhan Virus)
5. Going to the gym
6. Going for a walk in the park
7. Ordering lunch using food delivery
8. Walking past someone who has (COVID-19/Coronavirus/Wuhan Virus)
9. Sitting next to someone on the bus for 5 min who has (COVID-19/Coronavirus/Wuhan Virus)
10. Spending 30 min of close contact (e.g., conversation) with someone who has (COVID-19/Coronavirus/Wuhan Virus)
